# Controlling Dispersion Characteristics of Terahertz Metasurface

**DOI:** 10.1038/srep09367

**Published:** 2015-03-23

**Authors:** Shi-Wei Qu, Wei-Wei Wu, Bao-Jie Chen, Huan Yi, Xue Bai, Kung Bo Ng, Chi Hou Chan

**Affiliations:** 1 School of Electronic Engineering, University of Electronic Science and Technology of China (UESTC) 2006 Xiyuan Ave., Western-High Tech Zone, Chengdu 611731, China; 2 The State Key Lab of Millimeter Waves, City University of Hong Kong 83 Tat Chee Ave., Kowloon, Hong Kong, China

## Abstract

Terahertz (THz) metasurfaces have been explored recently due to their properties such as low material loss and ease of fabrication compared to three-dimensional (3D) metamaterials. Although the dispersion properties of the reflection/transmission-type THz metasurface were observed in some published literature, the method to control them at will has been scarcely reported to the best of our knowledge. In this context, flexible dispersion control of the THz metasurface will lead to great opportunities toward unprecedented THz devices. As an example, a THz metasurface with controllable dispersion characteristics has been successfully demonstrated in this article, and the incident waves at different frequencies from a source in front of the metasurface can be projected into different desired anomalous angular positions. Furthermore, this work provides a potential approach to other kinds of novel THz devices that need controllable metasurface dispersion properties.

Metamaterials have attracted much attention around the world since J. B. Pendry^1^ and D. Smith^2^ proposed the concept of negative permittivity and permeability, due to their many interesting phenomena which cannot be normally found in nature, e.g., negative or near-zero refractive index. When small inclusions in the three-dimensional (3D) metamaterial are placed into a two-dimensional (2D) pattern on a surface, the newly formed structure can also present some similar electromagnetic properties, called metafilm or metasurface. The behaviour of metasurfaces can be characterized by the electric and magnetic polarizabilities of their constituent inclusions, also similar to that of 3D metamaterials. Comparatively, metasurfaces have many inherent advantages of their own, e.g., less space occupation, possibility of lower loss and cost, and ease of fabrication. Therefore, the researches on metasurfaces are explosively expanded from microwave to optics in recent years^3^,^4^.

Terahertz (THz) technology has an increasing variety of applications^5^, such as non-destructive detection, security, biology and medical sciences. Benefited from recent technological innovation in photonics and nanotechnology, great progress has also been made in reflection/transmission-type THz metasurfaces. THz metasurfaces as absorbers are reported in some literature, e.g., THz absorbers with a broad and flat high absorption band by using I-shaped resonators^6^, polarization-insensitive broadband terahertz absorbers with multilayered cross^7^ or square patch elements^8^, and wide-angle absorber with Tungsten wire array^9^. As metasurface lens has an ultrathin profile, its aberration can be removed after a careful design^10^. Other metasurface based flat THz lenses, like an ultra-thin THz lens with an axial long focal depth^11^, have also been explored by some research groups. Moreover, a metasurface for anomalous reflection and refraction is obtained by using the unit cells with V-shaped inclusions, after the generalized laws of reflection and refraction are introduced firstly in Ref. 12. Based on similar ideas, interesting out-of-plane reflection and refraction of light by an anisotropic metasurface have also been reported^13^.

In Ref. 14, a metasurface is used to efficiently manipulate the wavefront of incident waves, as well as to convert the incident polarization into an orthogonal one. Recently, a metasurface with the capability of polarization conversion at THz frequencies are also reported in Ref. 15, and based on the same idea a metasurface based lens with anomalous refraction is presented. Meanwhile, the efficiency of a metasurface is studied by Jansen *et al.*^16^, and the quality factor can be significantly improved by introducing some asymmetries into the metasurface unit cell. Moreover, since the resonant property of the unit cell is quite sensitive to the surrounding material, it can also be applied in sensing applications^17^,^18^. Other attention is shifted to the tunable metasurfaces based on thermal effect^19^,^20^, for purposes like sensing or cloaking^21^. The so-called programmable beams of a metasurface under plane-wave incidence are presented in Ref. 22. However, poor agreements between simulated and experimental results can be found when the metasurface is experimentally illuminated by a horn, i.e., under the spherical wave incidence. Furthermore, the programmable devices are also unsuitable for THz operations due to the reasons like unavailability of THz switches. Active metasurfaces have also been explored for THz modulator uses^23^,^24^,^25^, and both spatial or phase modulation are demonstrated for prospective THz system applications based on a semi-insulating GaAs or silicon-on-sapphire wafer when they are biased by direct current (DC) voltage or pumped by a near-infrared laser pulses at 800 nm. Additionally, birefringent photonic device is generally bulky because it operates based on light path accumulation, but metasurface can help to solve this problem, e.g., the birefringent reflectarray metasurface in Ref. 26.

However, it is worth noting that the dispersion characteristics of the THz metasurface are often overlooked in many papers. In most cases, dispersion properties of metasurface are merely narrated, e.g., in Refs. 15 and 27, but without explanations on the reasons of beam/focus dispersion or any attempts to handle it. More importantly, studies on controlling the dispersion can scarcely be found although it is one of the most important properties of THz metasurfaces. It is found that a scanning beam within a small range of the metasurface is reported by Li, Y. B. *et al.*^28^, however, which operates based on holographic concept. Moreover, the scanning beams are caused by dispersive nature of the metasurface element, and no method to control the dispersion property of the holographic metasurface are mentioned therein. In this article, by using a specific kind of unit cells, a broadband dispersion controllable THz metasurface (DCTM) is demonstrated which can accurately project the incident THz waves of different wavelengths into different controllable anomalous directions. The device can be easily scaled downwards or upwards to microwave or optical frequencies after taking the material properties into account.

## Results

### THz Metasurface Configuration

**Figure 1:**
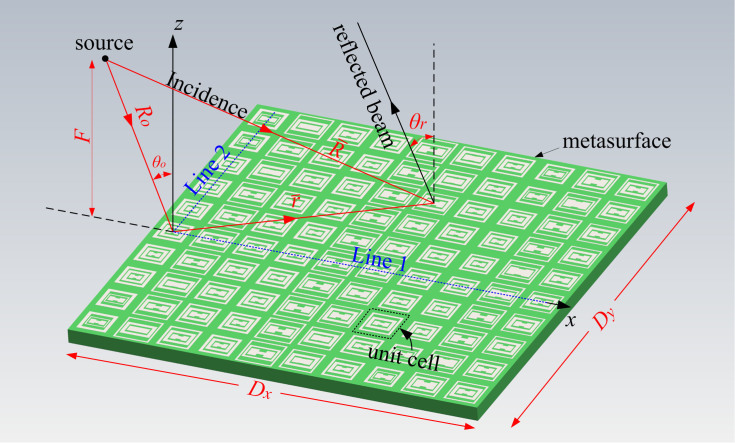
THz DCTM in the xy plane with a point source in the xz plane. For clarity, the metallic layer on the bottom is not shown. The origin of the coordinate system is located at the center of the first column of unit cells, and two referenced lines, i.e., Lines 1 and 2, have a common starting point but are oriented to the x and the y axes, respectively.

Figure 1 shows a DCTM on the top layer of a dielectric. There is a solid metallic layer on the bottom to reflect the transmitted waves, and therefore, the incident waves can interact with the metasurface twice, which means that more obvious interactions can be observed. Assume that a metasurface is located in the xy plane, a point source is placed in the xz plane by a distance *F* above the metasurface, and the incident angle from the source to the origin of the coordinate system is denoted by *θ_o_*. Meanwhile, at an arbitrary position 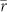 on the metasurface with a distance *R* to the source, the incident waves will impinge on the DCTM by an angle related to 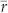.

The waves incident on the metasurface are expected to be reflected to an identical angle *θ_r_* with respect to the z axis at a given frequency *f*. As we know, the phase shift Φ of the metasurface unit cell is frequency dependent due to its dispersive nature. Meanwhile, the unit cells at different positions on the DCTM should be assigned by different phase shifts to compensate different spatial phase delay, meaning that the required phase shift Φ is also position dependent Φ(*f*, 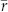). Additionally, *θ_r_* is a function of frequency because of the dispersive nature of the metasurface, i.e., *θ_r_* = *θ_r_*(*f*). Therefore, it can be derived that if the dispersion properties of the metasurface are expected to be controllable, e.g., an arbitrarily designed regular or anomalous *θ_r_* at any given *f*, the required phase distributions should satisfy the condition in Eq. (1):
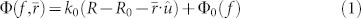
in which *k_0_* is the free-space wave number at *f*, Φ_o_(*f*) is the frequency-dependent phase shift of the unit cell at the origin of the coordinate system, and 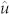 denotes the unit vector of the propagation direction of the reflected waves. Here, the physical dimensions of the DCTM are designed to be *D_x_* = 14.58*λ_0_* and *D_y_* = 11.67*λ_0_* at the center frequency *f_0_* = 250 GHz, composed of 50 × 40 unit cells. The source is placed at the location (−22.5*λ_0_*, 0, 25*λ_0_*) or (−27 mm, 0 mm, 30 mm), for considerations of measurements. The reflected beams are desired to be oriented to anomalous angles for the same reason, i.e., *θ_r_* = −35°, −25°, −15°, −10°, and −5° at 200, 225, 250, 275 and 300 GHz, respectively.

**Figure 2:**
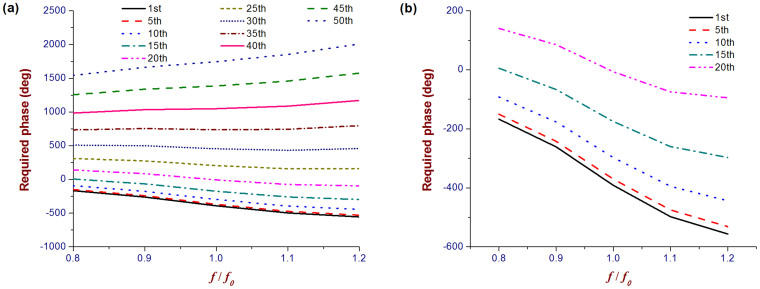
Designed reflection phases of the unit cells versus normalized frequency. Labels in the two figures are the numerical order of the unit cells from the starting point of the two referenced lines, i.e., the origin of the coordinate system. The horizontal axes represent the frequency normalized by the centre *f_0_* = 250 GHz. (a) A phase delay in degree, instead of a phase advance, for the unit cell on Lines 1 is always required and the curve with large numerical order becomes steeper. (b) The required reflection phase curve gradually becomes flatter and flatter when the numerical order of the unit cell on Line 2 goes larger.

To show the required phase by different unit cells on the metasurface, two representative lines along the x and y axes are chosen, i.e., Lines 1 and 2, both starting from the origin of the coordinate system. Figures 2a and 2b show the designed reflection phases at different positions of the two referenced lines, respectively, versus normalized frequency. A practically available Φ_o_(*f*) is chosen, as shown by the solid black curves, and Φ(*f*) at other positions of Lines 1 and 2 can be calculated according to Eq. (1). It can be seen from Figure 2a that the phase curve versus frequency becomes steeper and steeper as the unit cell on Line 1 is farther away from the starting point. Comparatively, the phase curve in Figure 2b becomes flatter for the unit cells farther away from the starting point of Line 2. These phenomena put severe limitations on the feasible sizes of the DCTM along the y-axis direction, which will be discussed later.

### Configuration and Characteristics of Unit Cell

**Figure 3:**
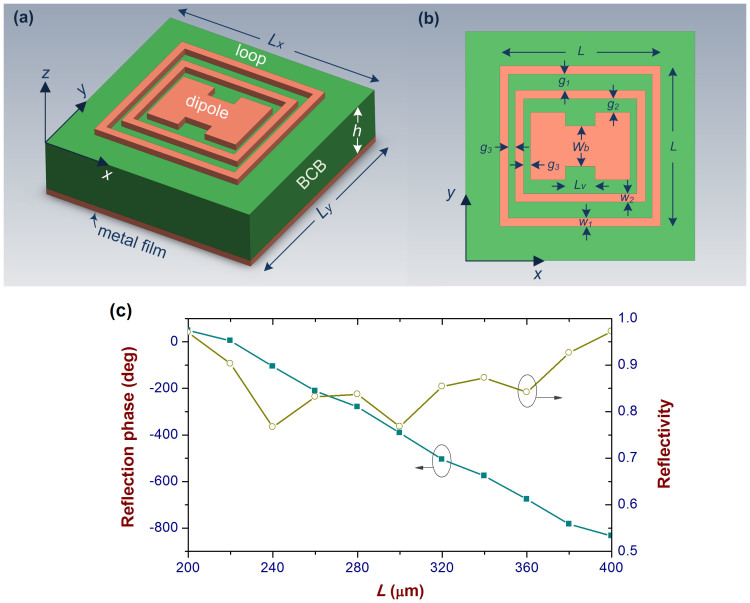
Unit cell geometry and its simulated properties. (a) 3D view. It is composed of a thin aluminum film, a BCB dielectric layer, and an aluminum pattern on the top. The total sizes of the unit cell are *L_x_* = *L_y_*, and *h* (fixed to be 120 μm in all cases). The 1 μm thickness of both aluminum layers are fixed in all cases of this paper. (b) Top view of the unit cell. There are two loops and an I-shaped dipole along the x axis. (c) The reflection phase in degree and reflectivity versus physical parameter *L* at 250 GHz. A linear phase curve and a large reflectivity are simultaneously obtained by properly choosing the physical parameters: *g_1_* = 35, *g_2_* = 20, *g_3_* = 9, *w_1_* = *w_2_* = 18.2, *L_x_* = *L_y_* = 500 in μm, *b* = 0.6 and *v* = 0.3. Note that the larger the reflectivity, the higher the efficiency of the DCTM is.

If the reflected beam dispersion of the metasurface is expected to be flexibly controlled, the property of the unit cell is one of the key factors. It must have the ability to present a variety of reflection phase curves with different ranges and different slopes when its physical size varies. Figure 3 shows the geometry of the employed unit cell which can meet the phase requirements of the 50 × 40 element DCTM. It is a three-layer structure, an aluminum film with a thickness of 1 μm as the bottom layer as well as a benzocyclobutene (BCB) layer with a thickness *h* and a refractive index 1.565 at the center, as shown in Figure 3a. Two loops and an I-shaped dipole on the top layer are made of 1 μm thick aluminum, each of which provides one resonance. Generally, one resonance can provide a phase range of over 300° and therefore the proposed unit cell can present a phase range as large as over 900°. Parameters of the structures on the top layer are given in Figure 3b, and another two are implicitly defined, i.e., *v* equal to *l_v_* over the total length of I-shaped dipole and *b* equal to *w_b_* over the total width of I-shaped dipole, for a proportional size variation of the I-shaped dipole to control the reflection phase.

According to our studies, if a linear reflection phase curve is desired, mutual coupling among the three components should be carefully tuned. Reducing the two gaps with a size *g_3_* in Figure 3b can significantly enhance the electric coupling strength between three components, which will push the three resonances closer and lead to a linear reflection phase curve. However, if *g_3_* is too small, the reflection phase range would be reduced because the three resonances go too close to each other. Comparatively, the two gaps with dimensions *g_1_* and *g_2_* determine the magnetic coupling between three components, which can also enhance the reflection phase range but at the cost of reduced reflectivity.

Figure 3c gives the reflection phase curve and reflectivity of the metasurface unit cell with the parameters shown in the caption of the figure under normal incidence. It can be seen that the unit cell is able to present a phase range of over 900° with good linearity as parameter *L* is changed from 200 to 400 μm at 250 GHz. Meanwhile, the reflectivity of such a structure is over 0.75 as *L* varies within the same range. Three dips on the reflectivity curve denote the three resonances. The electric current distributions on three components of the unit cell can be found in Section S.1 in the Supplementary Information, which can provide more information about the three resonances. In the whole interested frequency band of 200 ~ 300 GHz, the unit cell with the given parameters can cover a reflection phase range of over 400° (See Section S.2 in the Supplementary Information). Meanwhile, the reflectivity across the whole frequency band is over 0.75 which is benefited from optimization of electric and magnetic couplings between three components on the top layer of the unit cell. Furthermore, the slope and range of its reflection phase can be flexibly tuned as shown in Section S.3 in the Supplementary Information. Three figures therein give us clear information that the phase required by the metasurface to control the dispersion properties can be satisfied by the employed unit cells.

### Numerical and Experimental Results

**Figure 4:**
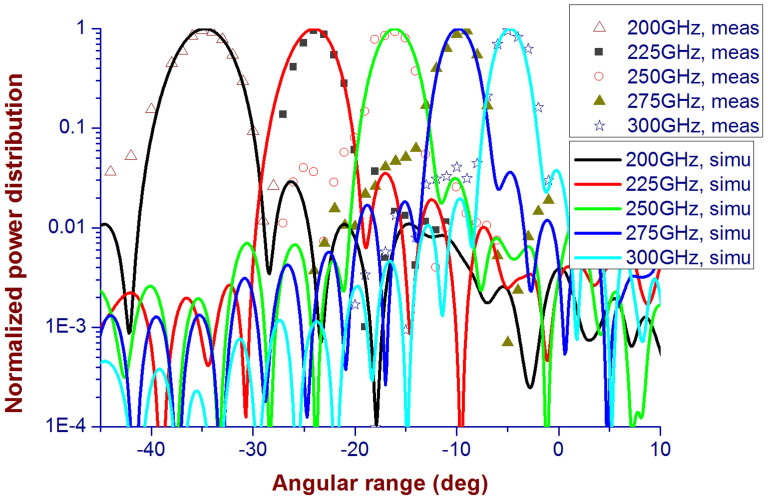
Measured and simulated results of the DCTM prototype. In the figure, the power distribution within an angular range from −45° to 10° is shown. All measured and simulated results are normalized to their maximum values. The measured results are shown by different types of markers at different frequencies, and the simulated ones are by the smooth curves. The simulated results at five frequencies are given, i.e., 200, 225, 250, 275, and 300 GHz, while the measurements are performed with an angular step 1° by two pairs of the OML extenders from 140 to 220 GHz (for 200 GHz measurements) and from 220 to 325 GHz (for measurements at 225, 250, 275 and 300 GHz), respectively.

Beam positions of the fabricated DCTM at 200, 225, 250, 275, and 300 GHz are originally designed at −35°, −25°, −15°, −10°, and −5°, respectively. The full-wave simulations show that the DCTM presents a slight beam shift by around 1° from the designed values, as given in Figure 4, because of the numerical errors in the simulated phase of the unit cell.

From the figure, we can see that measurements agree reasonably well with the simulations near both sides of the position with maximum electric field intensity. There are slight differences at the positions far away from the peak, which is attributed by the errors in fabrications and measurements, as explained in Section S.7 in the Supplementary Information. The proposed idea of controlling the dispersion properties of the metasurface has been proved both numerically and experimentally in this article.

## Discussion

Although controllable anomalous reflection beam angles of the THz metasurface versus different frequencies are successfully demonstrated, there are actually significant limitations on the maximum achievable sizes in the x- and y-axis directions due to the following reasons. For an assumed phase curve Φ_o_(*f*), the required phase curves of the unit cells on Lines 1 and 2 in Figure 1 are given in Section S.4 in the Supplementary Information to show the conditions required by controlling dispersion properties of the metasurface. From Fig. S5a, it can be seen that the required phase curve versus frequency becomes steeper and steeper when the position of the unit cell is farther away from the starting point of Line 1. Fortunately, a phase delay instead of a phase advance is always required. However, the achievable slope of the reflection phase for a given unit cell geometry is generally limited within a reasonable range. Otherwise, if the unit cells are placed in the -x-axis direction, the required phase curve would exhibit a positive slope, which is actually unachievable in physics without significant reflectivity degradation. Fig. S6a shows the required reflection phase of the unit cells on Line 2. It can be seen that the slope of the required phase curve is changed from a negative to a nonphysical positive value as the unit cell is farther and farther away from the starting point of Line 2. Moreover, nonlinearity of the required phase curves along Lines 1 and 2 in Figs. S5b and S6b brings more restrictions on the achievable DCTM sizes.

Note that, if we find the first order derivative of Φ(*f*, 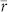) in Eq. (1) with respect to independent variable *r*, Eq. (2) can be obtained:
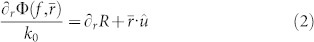
Actually, when the source is placed by an infinite distance from the metasurface, indicating the plane-wave incidence, *R* will be independent on 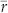, and Eq. (2) can be simplified to the generalized law of reflection proposed in Ref. 12. Therefore, what we are studying is more general than the case in Ref. 12. For the plane-wave incident case, the phase required by each unit cell is actually independent on the y-axis position, and then no limitation is put on the dimension of the DCTM along y-direction. Therefore, only the desired properties of unit cells along Line 1 are studied, as shown in Fig. S7 in the Supplementary Information. It can be concluded that the design of such a DCTM in the plane-wave incident case is much easier than the general case studied in this article. In other word, the difficulties are gradually decreased as the source is put farther and farther away from the DCTM.

Although the metasurface in Ref. 28 can also present frequency scanning beams, only the phenomenon of dispersive beams are mentioned, which is just a natural characteristic of broadband devices, and the dispersion properties cannot be controlled therein. Secondly, the operating principles of both devices are totally different. The holographic concept is employed in Ref. 28, but the multiple frequency phase matching method is used in this work, which means that the dispersive nature of the proposed THz metasurface is purposely controlled.

## Methods

### THz metasurface design

To successfully implement the proposed concept of the DCTM, a database of the unit cell with different physical parameters is firstly built. Since not all parameters are critical to tune the reflection phase, only five are chosen to map the physical sizes of the unit cell to the achieved reflection phase, as given in Section S.5 in the Supplementary Information. In the multi-dimensional database, the index of each element indicates the physical size of the unit cell and the value corresponds to the reflection phase. Then, the DCTM can be designed based on the database after the source position is provided.

To design the DCTM, the unit cells with proper physical dimensions are carefully selected from the well-prepared database by multi-frequency phase matching method, i.e., simultaneously matching the achievable to the desired reflection phases of each element at 200, 225, 250, 275, and 300 GHz^29^,^30^. In this process quite strict demands are set on the element performances which, however, can simplify the metasurface design in return. The method to control the dispersion of a metasurface can be also extended to other kinds of applications, e.g., a meta-lens with a controllable focal position versus frequency.

### Fabrication

**Figure 5:**
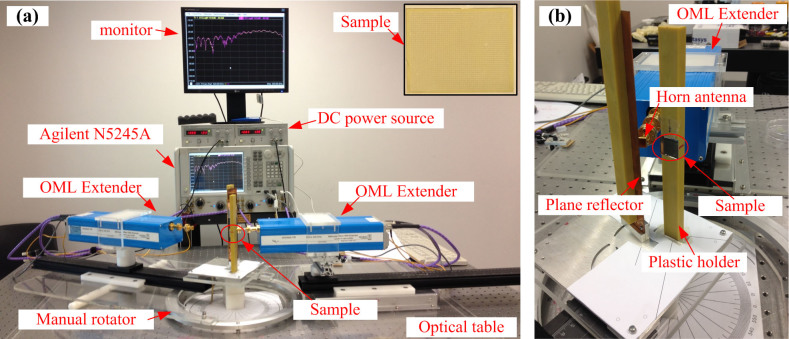
Photographs of measurement setup. (a) Full view of the setup. A vector network analyzer (Agilent N5245A) with a maximum operating frequency up to 50 GHz is located at the center of the photograph. For THz measurement, two pairs of OML extenders (Model V05VNA2-T/R and V03VNA2-T/R) are used to extend the operating frequency up to 140 ~ 220 GHz and 225 ~ 325 GHz. The two DC power sources are used for the two OML extenders. The monitor is used to display the measured data more clearly. The left OML extender with a receiving horn antenna is placed on a manual rotator to measure the electric fields within an angular range, while the right one and the horn antenna are used for transmitting purpose. The two OML extenders, rotator, and DCTM prototype are placed on an automatically horizontal optical table. The inset in the upper-right corner shows photograph of the full DCTM prototype. (b) Local view of the prototype. The DCTM prototype installed on a plastic holder is clearly shown along with the transmitting horn antenna and the planar reflector.

The DCTM was fabricated by standard micro-fabrication method in the City University of Hong Kong. First, polymer BCB from Dow Chemical Company, was spin-coated and cured (at 270°C for 2 hours) onto a flat aluminum plate. As previously stated, the thickness of the BCB layer was set at ~120 μm and determined by the spinning speed and time (1000 rpm, 2 min, spin coating for 3 times). Then, a ~1 μm thick aluminum film was deposited onto the BCB layer by thermal evaporation. Finally, the aluminum pattern was fabricated by photolithography process followed by aluminum wet etching. Photographs of the fabricated DCTM prototype can be found in the inset of Figure 5a and Fig. S8 in the Supplementary Information.

### Measurement setup

**Figure 6:**
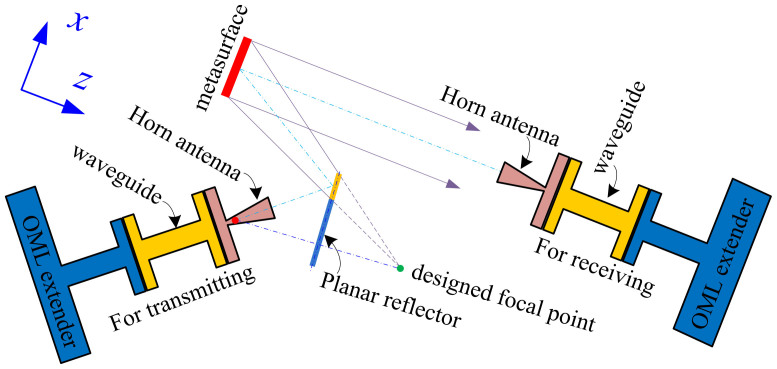
Schematic of the measurement setup of the metasurface. The DCTM denoted in red is initially designed to have a focus shown by the green dot. But for measurement considerations, a planar reflector is employed to produce a virtual focus shown by the red dot. The dash-dot lines with light color shows the optical axis of the conceived measurement setup, while the one in blue shows the mirror effect of the focus by the planar reflector. In the figure, the area with a positive x- and a positive z-coordinate is denoted by a positive *θ* while the area with a negative x- but a positive z-coordinate is by a negative *θ*.

To measure the DCTM, a measurement setup as shown in Figure 6 is conceived, after considering the size of the measurement facilities, such as OML extenders, horn antennas, and rectangular waveguides etc. The OML extenders are quite huge compared to the fabricated DCTM prototype, and a planar reflector must be used to produce a mirror image of the designed focus of the DCTM to flexibly measure the THz power distributions within an angular range in the xz plane. It should be noted that the phase center of the transmitting horn antenna should be placed at the virtual focal position so that the spherical waves from the horn can be equivalently emitted from the virtual focus. Meanwhile, the reflection angle *θ_r_* of the DCTM is designed to be anomalous for two reasons. The first is to avoid direct power leakage from the transmitting horn antenna to the receiving horn during experiments. In the figure, the emitted waves from the horn antenna are reflected by the yellow part of the planar reflector and then exactly impinge on the DCTM. The extended part in light blue is to avoid direct power leakage into the angular range with a negative *θ* from the transmitting horn antenna to the receiving one for power distribution measurements. The second is to remove the interference of the scattered waves by other structures like the plastic holder etc.

The receiving horn is placed at 0.4 m from the center of the DCTM, which is also the maximum distance available in our measurement setup, to avoid physical interference between the transmitting and the receiving modules. Finally, by rotating the receiving horn antenna with respect to the center of the DCTM, the THz power distribution on an arc can be measured. Photographs of measurement setup are shown in Figure 5. Each OML extender is connected to one port of Agilent N5245A network analyzer by coaxial cables, and the measured angular range is limited by the length of these cables.

## Author Contributions

S.W.Q. and C.H.C. are responsible for theoretical design based on an original idea by S.W.Q., and wrote the manuscript. S.W.Q., W.W.W. and Y.H. performed the numerical simulations. B.J.C. fabricated the samples. X.B. and K.B.N. performed the measurements.

## Supplementary Material

Supplementary Information Supplementary Information
