# Supplementary material for: Controlling Dispersion Characteristics of Terahertz Metasurface
Source: Sci Rep. 2015 Mar 23;5:9367. doi: 10.1038/srep09367 (PMC4369731; doi:10.1038/srep09367)
Supplement: Supplementary Information [file srep09367-s1.pdf]

SUPPLEMENTARY INFORMATION

CONTROLLING DISPERSION CHARACTERISTICS OF TERAHERTZ METASURFACE

Shi-Wei Qu, Wei-Wei Wu, Bao-Jie Chen, Huan Yi, XueBai, Kung Bo Ng, and Chi Hou Chan

S.1 Current distributions on the Unit Cell

The current distributions on a unit cell with different parameter L at 250GHz are presented Fig. S1 for references. Note that the two loops and I-shaped dipole are designed to be proportionally changed with respect to L, for considerations of controlling the reflection phase curve. As L is changed, other parameters are fixed as given in the caption of Fig. S1. It is also clear that as L is increased from 240 to 360μm, the currents are concentrated on the outer loop, the inner loop, and the edges of the I-shaped dipole, respectively. The current distributions in Fig. S1 indicate that the three components can resonate individually at 250GHz by properly changing their physical sizes.

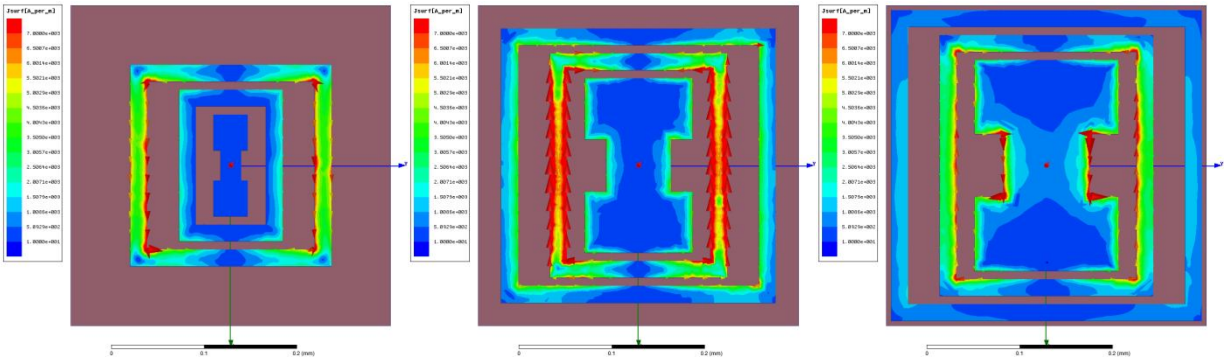

(a) (b) (c)

Fig. S1. Electric current distributions on the loops and I-shaped dipole at 250GHz as (a) L = 220μm, (b) L = 290μm, and (c) L = 340μm. Note that the three components are proportionally changed with respect to parameter L. Other parameters are as follows: g_1 = 35, g_2 = 20, g_3 = 9, w_1 = w_2= 18.2, L_x = L_y = 350, in μm, b = 0.6 and v = 0.3.

In Fig. S2, the electric current distributions on the three components with fixed dimensions are given at 200, 250 and 300GHz, which are the lower edge, middle, and upper edge of the interested frequency band, respectively. It is clear that the currents are gradually concentrated onto the central part of the unit cell as frequency increases, because smaller component resonates at higher frequency. A significant part of the currents at 200GHz are distributed on the larger loop resonator, and the currents at 250 and 300GHz are dominated by the two loop resonators, the inner ring and the I-shaped dipole, respectively.

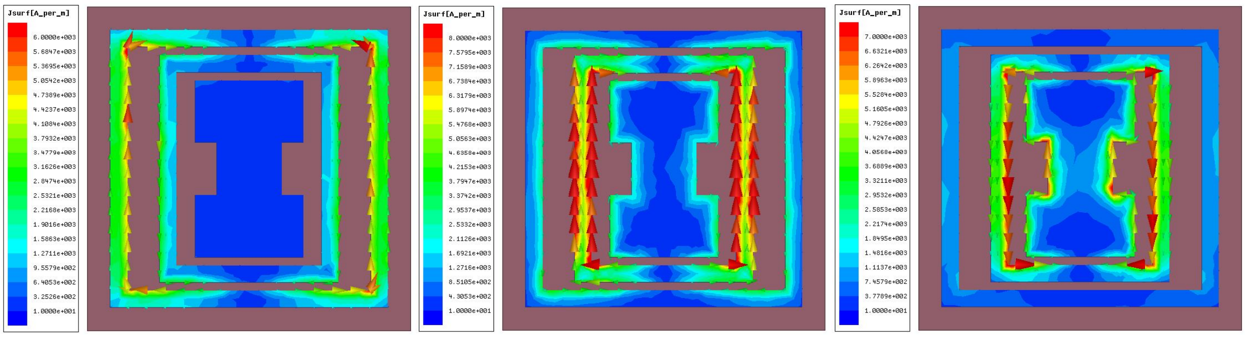

(a) (b) (c)

Fig. S2. Electric current distributions on the loops and I-shaped dipole at (a) 200, (b) 250 and (c) 300GHz. The currents on the three components with fixed dimensions but at different frequencies are given here, compared to those in Fig. S1.The physical parameters are as follows: g_1 = 35, g_2 = 20, g_3 = 9, w_1 = w_2= 18.2, L = 300, L_x = L_y = 350, in μm, b = 0.6 and v = 0.3.

S.2 Broadband Property of the Unit Cell

Reflection phase and reflectivity of the proposed unit cell versus physical parameter L are given at 250GHz in the main text. Its broadband characteristics are also critical for the dispersion controllable terahertz (THz) metasurface (DCTM) design. In Fig. S3, the reflection phase and reflectivity versus frequency are shown. For clarity, the frequency is normalized by the center frequency, f_0 = 250GHz. Dimensions of the unit cell in this figure can be found in the caption of Fig. S3. A linear phase curve with a range over 300° is also observed and the reflectivity is still over 0.8 within a frequency bandwidth of 40%. Actually, larger reflection phase range versus frequency can be obtained by properly tuning the physical parameters.

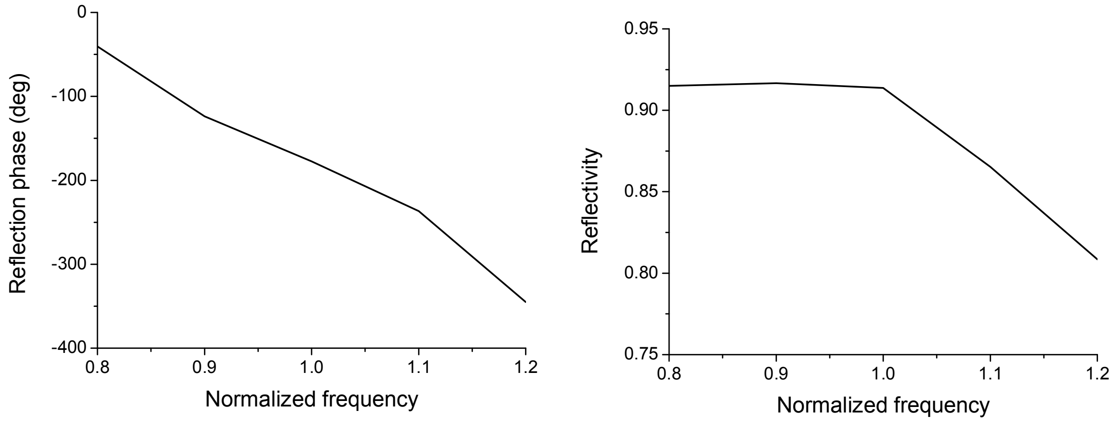

(a) (b)

Fig. S3. (a) Reflection phase and (b) reflectivity of the unit cell versus normalized frequency. The physical parameters are as follows: g_1 = 35, g_2 = 20, g_3 = 9, L = 250, w_1 = w_2 = 18.2, L_x = L_y = 350, in μm, b = 0.6, and v = 0.3.

S.3 Control of Range and Slope of Phase Curve

To control the dispersion of the DCTM, the unit cell is extensively studied to explore its electromagnetic properties. Fig. S4 shows the control of the slope and range of the reflection phase by combining different physical parameters. In Fig. S4 (a), there are 14 curves in total which can be divided into three groups. Each group of the phase curves has a common cross point, meaning that different slopes as well as different reflection phase ranges can be obtained as parameter w_2 is changed from 10 to 30 μm. Meanwhile, three groups have three horizontally separated cross points which provide more solutions to control the slope and range of the reflection phase. In Fig. S4 (b), several examples are shown to simultaneously control the slope and range by the physical parameter b, and comparatively only the phase range is tuned in Fig. S4 (c). In Fig. S4 (c), a set of parallel and linear phase curves can be obtained as w_1 varies. The three figures mentioned above give us clear information that the required phase by the DCTM to control the dispersive properties can be satisfied by the proposed unit cells.

The operating principle of the controllable range and slope of the reflection phase is as follows. There are three resonant components in the unit cell, between which the mutual coupling dominates the range and slope of the reflection phase. Stronger electric coupling between three components, which is determined by the two gaps with a size g3 in Figure 3b, will push the three resonances closer, leading to a linear reflection phase curve. The phase range is controlled by the separation of the three resonances. For a very small g_3, strong mutual coupling will make the three resonances indistinguishable, resulting in the reduced reflection phase range. Comparatively, the magnetic coupling between three components is determined by the two gaps with dimensions g_1 and g_2. Improving the magnetic coupling can also enhance the reflection phase range, but at the cost of reduced reflectivity due to stronger concentration of the electric current distributions on the three components.

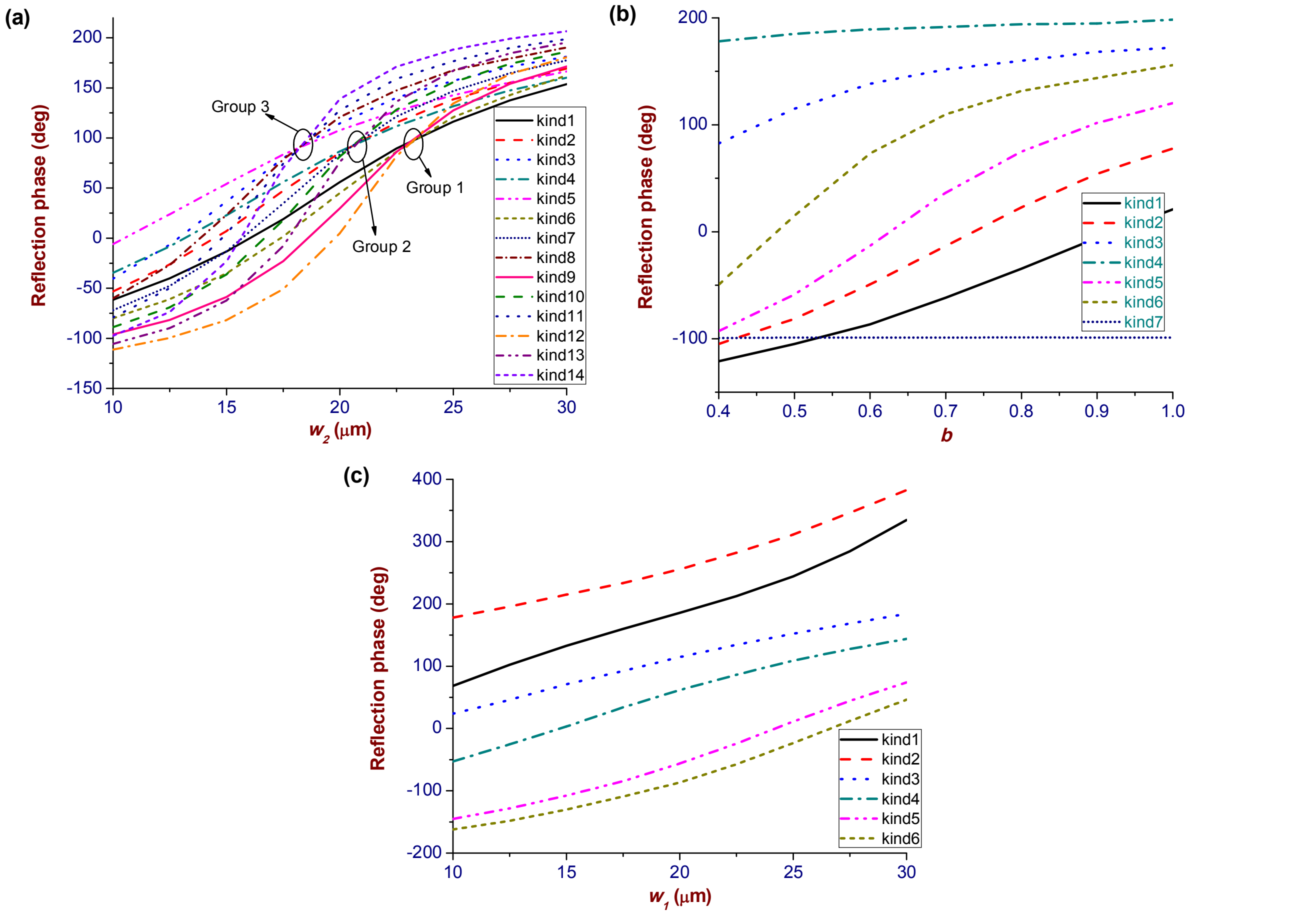

Fig. S4. Demonstration of controlling slope and range of the reflection phase at 300GHz versus physical parameters (a) w_2 and (b) b, as well as (c) independently controlling phase range by parameter w_1.

S.4 Phase Requirements in Metasurface Grating Design

With the point source placed at position (0, 0, F) and $F = D_x = D_y/2 = 25\lambda_0$ at the center frequency f_0, we assume that the metasurface is discretized into 50 × 100 unit cells along the x- and y-axis directions, which is larger than the prototype in the main content for better understanding the connection between the required phase and the position of the element on the metasurface. Both Lines 1 and 2 are labeled as segments 1 to 50 from a common starting point, i.e., the origin of the coordinate system. The reflection beam is desired to be oriented to θ_r = -35°, -25°, -15°, -10°, and -5° at the normalized frequency f/f_0 = 0.8, 0.9, 1, 1.1 and 1.2, respectively.

Fig. S5 (a) shows the required reflection phases at different positions of Line 1 versus the normalized frequency. The solid black curve is chosen as $\Phi_o(f)$. Fig. S5 (b) gives the required reflection phases at different frequencies versus numerical order of the unit cell on Line 1, and the curves therein are obviously nonlinear which creates more difficulties on the DCTM design. Therefore, there is actually a limitation on the maximum achievable size of the DCTM in the x-axis direction.

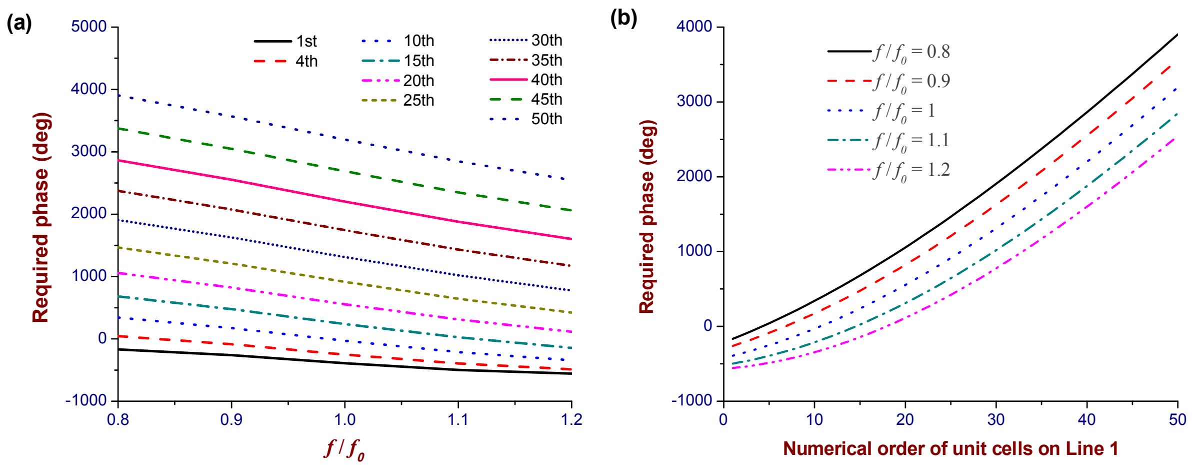

Fig. S5. Required reflection phase of the unit cells on Line 1 versus (a) frequency and (b) numerical order of the unit cell. The label in Fig. S5 (a) is the numerical order of the unit cell from the starting point of Line 1. The horizontal axes of Figs. S5 (a) and (b) are the normalized frequency and the numerical order of the unit cells, respectively.

In Fig. S6 (a), the required phase of the unit cell at different position on Line 2 versus normalized frequency is shown, as the phase curve of the first unit cell is assumed beforehand. This transition point is located at the 33rd unit cell, and the slope has already become positive from a negative value. This conclusion can also be proved by the data in Fig. S6 (b). For the curve of different frequencies, there is a common cross point at the 33rd unit cell, across which the slope of the required phase curve versus frequency is actually reversed.

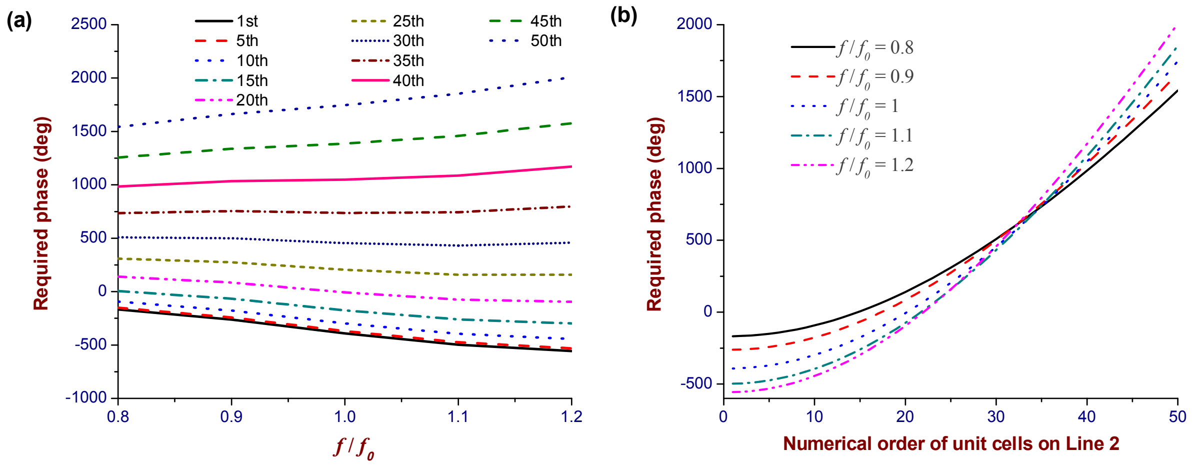

Fig. S6. Required reflection phase of the unit cells on Line 2 versus (a) frequency and (b) numerical order of the unit cell. The label in Fig. S6 (a) means the numerical order of the unit cell from the starting point of Line 2.

For the plane-wave incident case, the required phase by the unit cell is actually independent on the size of the DCTM along the y axis. Therefore, only the desired properties of unit cells along Line 1 are shown in Fig. S7. It can be seen from Fig. S7 (a) that the required phase curve for the unit cell far away from the starting point of Line 1 becomes

steeper, but no phase advance versus frequency is observed. Meanwhile, the slope of each curve is much smaller than that in Fig. S5 (a), and the total required reflection phase range is also much smaller. In Fig. S7 (b), the phase curves are linear instead of the nonlinear ones in Fig. S5 (b). It is obvious that design of such a DCTM in the plane-wave incident case is much easier than the general case studied above.

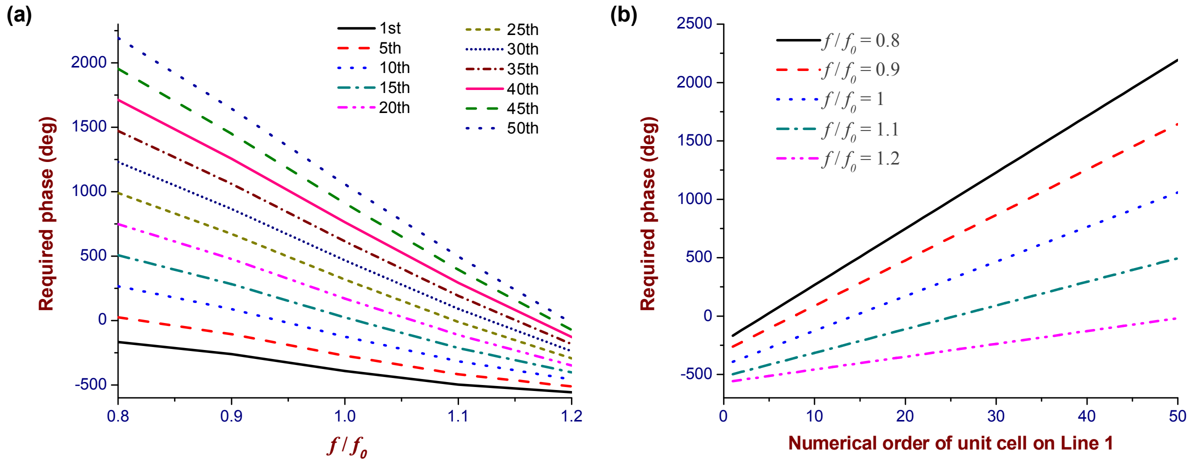

Fig. S7. For the plane-wave incident case, the required reflection phase of the unit cells on Line 1 versus (a) frequency and (b) numerical order of the unit cell. The phase curve of the first unit cell is the same to that in Fig. S5. The phase curves in Fig. S7 (b) become linear, instead of the nonlinear ones in Fig. S5 (b).

S.5 Reflection Phase Database

A database of the reflection phase of the unit cell is firstly built to map the physical sizes to the reflection phase at 200, 225, 250, 275 and 300GHz, respectively. The five parameters and the discretized steps are shown in Table IV. Here the dimensions of the unit cells are fixed to be $L_x = L_y = 350$μm, when taking the oblique incidence on the metasurface into account. The first four is discretized by a step of 5μm to reduce the required computational time in the full-wave simulations. After a careful parametric sweeping, an interpolation process is performed to obtain more detailed phase values with a step size of 0.5μm for the first four parameters, and parameter b is discretized by a step of 0.01 within the interpolation process. Then, a six-dimensional database has been established, in which the index of each element indicates the physical sizes of the unit cell and the value of that element corresponds to the reflection phase, as mentioned in the main content.

Table IV. Five swept parameters to build the database of reflection phase at 200, 225, 250, 275 and 300GHz.

Parameters	L (μm)	w_1 (μm)	w_2 (μm)	g_1 (μm)	b
Values	240 ~ 320	10 ~ 30	10 ~ 30	20 ~ 55	0.4 ~ 1
Step	5	5	5	5	0.1

S.6 Fabricated Prototype

Photographs of the fabricated metasurface prototype are shown in Fig. S8. Its total sizes are 17.5 × 14 mm^2 in the x- and y-axis directions. From the zoom-in view in the inset of Fig. S8, it can be seen that there are still some defects in a small portion of unit cells, which are one of the reasons causing differences between simulations and measurements.

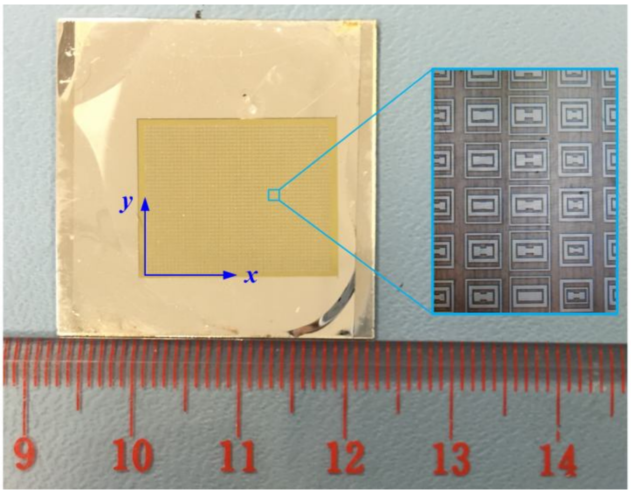

Fig. S8. Fabricated DCTM prototype and the zoom-in view. A ruler is placed beside the fabricated prototype for reference. The aluminum plate with very small roughness like a mirror can be clearly seen.

S.7 Error Analysis

There are many possible errors causing the discrepancies between measurements and simulations. The most significant are as follows.

1. Inevitable numerical errors when building the database of reflection phase and full-wave simulations of the whole DCTM.
2. Non-perfect prototype of the DCTM due to fabrication tolerance. Actually, several samples were fabricated and the best one is selected for measurements.
3. Installation errors of the prototype. Since it is placed on a plastic holder, position errors in three dimensions will also be introduced during installation. They are actually noticeable because of very small wavelength at THz frequency.
4. Errors in measurement setup, e.g., misalignment of the phase center of the transmitting horn antenna against the virtual focus of the DCTM, pre-designed position error of the virtual focus caused by deformation of the planar reflector, misalignment of the DCTM against the center of the arc on which the electric field intensity is measured.
5. Errors during measurements, e.g., scattering by the edges of the plastic holder and the planar reflector in Figure 6 of the main content.
